# A Lower Irradiation Dose of 308 nm Monochromatic Excimer Light Might Be Sufficient for Vitiligo Treatment: A Novel Insight Gained from In Vitro and In Vivo Analyses

**DOI:** 10.3390/ijms221910409

**Published:** 2021-09-27

**Authors:** Yasutaka Kuroda, Lingli Yang, Sylvia Lai, Jiao Guo, Tetsuya Sayo, Yoshito Takahashi, Daisuke Tsuruta, Ichiro Katayama

**Affiliations:** 1 Department of Pigmentation Research and Therapeutics, Graduate School of Medicine, Osaka City University, Osaka 5450051, Japan; kuroda.yasutaka@kao.com (Y.K.); s-lai@l.wdb-eu.com (S.L.); guojiao1986@gmail.com (J.G.); sayou.tetsuya@kao.com (T.S.); takahashi.yoshito@kao.com (Y.T.); espaikoffice@gmail.com (I.K.); 2 Biological Science Research Laboratories, Kao Corporation, Odawara 2500002, Japan; 3 Department of Dermatology, Graduate School of Medicine, Osaka City University, Osaka 5458585, Japan; dtsuruta@med.osaka-cu.ac.jp

**Keywords:** pigmentation, excimer light, ultraviolet, keratinocyte, inflammasome

## Abstract

A 308 nm monochromatic excimer light (MEL) is widely used to treat patients with vitiligo. However, dose optimization still needs to be clarified. This study aimed to obtain objective evidence regarding various doses of MEL irradiation, induced cell level changes in vitro, and skin level alterations in vivo. Cultured human keratinocytes were irradiated with MEL using various doses. After irradiation at low doses, stem cell factor, endothelin-1, and glycoprotein nonmetastatic melanoma protein B, factors that activate and protect melanocytes, were found to be significantly elevated in keratinocytes. After irradiation using medium and high doses, inflammatory cytokines were induced. The amount of ATP released and the level of inflammasome activation, which are known to be related to interleukin-1β activation, were also increased. The back skin of guinea pigs and mice were irradiated with MEL at varying doses. After irradiation, an increase of epidermal melanin and epidermal melanocytes was confirmed, using the minimal erythemal dose or less. In rhododendrol-induced leukoderma guinea pigs, a much lower dose of MEL irradiation was effective, when compared with the effective dose for control guinea pigs. Our results suggest that a lower irradiation dose of MEL might be sufficient and more suitable for repigmentation in vitiligo treatment.

## 1. Introduction

Skin locates at the interface between external and internal environments. It was established as an important neuro-endocrine-immune organ because it can sense the environment and regulate the local and global homeostasis [1,2]. Ultraviolet (UV) radiation is a critical environmental factor influencing skin physiology. UV-sensitive keratinocytes, melanocytes and other cutaneous cells can sense UV and translate it into defined biological responses, e.g., the synthesis and release of melanin, neurohormones and cytokines [3,4]. UVB (280–315 nm) induces skin tanning by increased synthesis of melanin following the stimulation of melanogenic factors in melanocytes [5,6]. Melanogenesis is regulated directly or indirectly through various hormone stimulations, and it is strongly regulated via interactions with neighboring cells in the skin [2,7,8]. A lot of melanogenic paracrine factors produced by UVB exposed keratinocytes were identified [9]. Stem cell factor (SCF) and endothelin-1 (ET-1) are key paracrine melanogenic factors induced by UVB, and these factors bind to their corresponding receptors expressed on melanocytes [10].

Vitiligo is a commonly acquired pigmentation skin disorder, characterized by depigmented patches resulting from loss of epidermal functional melanocytes. It affects approximately 0.5–2% of the world population, without any sex, age, or racial differences [11]. It is one of the most psychologically devastating skin diseases because it presents a disfiguring contrast of skin colors between healthy and lesional areas. Therefore, vitiligo patients have a significant reduction in their quality of life [12].

The exact pathogenesis of vitiligo remains unknown. Several hypotheses including autoimmunity, neuronal stress, oxidative stress, autocytotoxicity, and melanocytorrhagy were proposed [13,14]. The treatment choices for vitiligo include topical and systemic immunosuppressants, surgery, autologous melanocytes transplantation, cosmetic camouflage, and phototherapy [13,15,16].

Although several treatment choices are available for vitiligo patients, no definite cure has yet been developed [16]. Phototherapy is used widely with success and is currently considered as the first-line choice for vitiligo treatment. It includes the conventional treatment of topical or systemic psoralen plus ultraviolet A (PUVA) photochemotherapy, the current standard treatments involve narrow band UV-B (NBUVB), which is used for generalized vitiligo, and a recent novel treatment using a 308 nm monochromatic excimer laser (MEL), which is used to treat localized disease [16,17]. Historically, until a few years ago, topical/systemic PUVA was the most popular treatment and was considered the gold standard treatment for vitiligo worldwide. However, it is now thought to be only moderately effective for vitiligo, with adverse side effects including gastrointestinal irritation, phototoxicity, eye damage, and long-term carcinogenic risk [18,19]. In the past few years, NBUVB with a peak emission at 311–313 nm has emerged as the main and initial choice for vitiligo phototherapy. It is more effective and better tolerated than PUVA, there is no need for ocular protection, there are no adverse systemic side effects and rare phototoxic reactions, and there is no increase in the incidence of skin cancer [18,19]. However, it is difficult to achieve complete repigmentation in most cases, and it requires several months to obtain a therapeutic response [18,19]. Most recently, targeted phototherapy systems involving MEL have shown improved efficacy for the treatment of vitiligo [20,21]. The monochromatic radiation wavelength (308 nm) is close to that used in NBUVB phototherapy. It has some advantages over NBUVB phototherapy, including faster and better repigmentation, a shorter treatment duration, and more limited side effects. Another important advantage is that it delivers high intensity light exclusively to the localized affected depigmented areas, so exposure of the neighboring healthy skin is avoided, which lowers the cumulative irradiation dose [19,20,22].

In the treatment of vitiligo, MEL phototherapy is presently one of the most widely used new therapies [12]. However, during phototherapy, it is known that suitable irradiation promotes repigmentation of the vitiligo skin, and excessive irradiation induces acute and long-term adverse effects, which include skin erythema, xerosis, pruritus, blistering, hyperpigmentation, photoaging, and photocarcinogenesis [23]. These adverse effects increase with the cumulative dose. Therefore, a precise assessment of the relationship between irradiation dose and risk is required.

However, there is still little evidence and no consensus regarding the optimal dosimetric protocol for targeted phototherapy using MEL for the treatment of vitiligo [17,24]. To the best of our knowledge, with regard to vitiligo treatment, there is still no report about the use of MEL for cultured skin cells and experimental animals. Thus, in this study, we determined the optimal dose for the effective and safe vitiligo treatment, using both in vitro and in vivo analyses.

## 2. Results

### 2.1. The Nontoxic Dose of MEL Irradiation Induced Expressions of Stem Cell Factor (SCF), Endothelin-1 (ET-1), and Glycoprotein Nonmetastatic Melanoma Protein B (GPNMB) in Cultured Human Skin Epidermal Keratinocytes

Melanocyte function is strongly regulated by interactions with various hormones and cytokines [2,7,8]. Regarding neighboring cells in the skin, many melanogenic paracrine factors produced by keratinocytes have been identified [9]. SCF and ET-1 are key paracrine melanogenic factors induced by UVB. These factors bind to their corresponding receptors expressed on melanocytes and are responsible for melanocyte proliferation and migration to the skin epidermis in vivo [10,25,26,27]. In addition, they were found to be protective factors against oxidative stress in melanocytes (Wang and Kuroda et al., Submitted).

**Figure 1:**
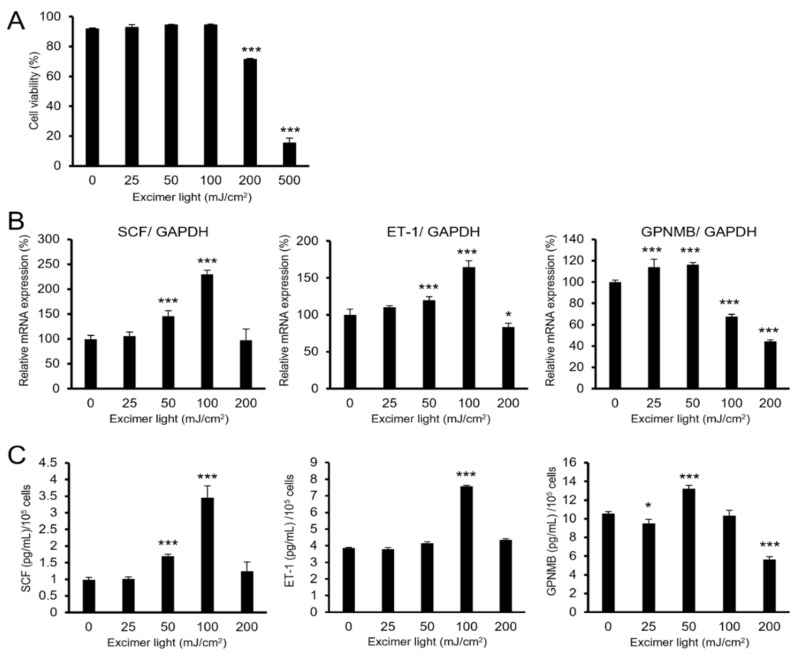
Monochromatic excimer light (MEL) irradiation with a nontoxic dose induced stem cell factor (SCF), endothelin-1 (ET-1), and glycoprotein nonmetastatic melanoma protein B (GPNMB) in PSVK1 keratinocytes. (**A**) Cell viability of PSVK1 keratinocytes after 24 h radiation with the indicated dose of MEL. (**B**) Relative mRNA expression of SCF, ET-1, and GPNMB after 24 h of irradiation with the indicated dose of MEL. (**C**) Protein expressions of SCF, ET-1, and GPNMB in cultured supernatants using an ELISA assay after 48 h of irradiation with the indicated dose of MEL. Data are shown as the means ± SD, * *p* < 0.05; *** *p* < 0.01; one-way analysis of variance followed by Dunnett’s test vs. 0 mJ/cm^2^.

To determine whether and how MEL irradiation affects these keratinocyte-derived repigmentation-related factors, the mRNA expression and released protein levels were measured after MEL radiation in the PSVK1 human keratinocyte cell line. Using cell viability analyses, the subtoxic and toxic doses of MEL irradiation were found to be <200 mJ/cm^2^ and ≥200 mJ/cm^2^, respectively, in monolayer cultures of PSVK1 cells (Figure 1A). Therefore, the cells were subsequently irradiated with 0–200 mJ/cm^2^ of excimer light. Expression of SCF and ET-1 mRNA and protein was induced with a peak at 100 mJ/cm^2^ (Figure 1B,C), while the expression of GPNMB was induced at a lower dose of 50 mJ/cm^2^. These results suggested that SCF, ET-1, and GPNMB were upregulated at less than toxic doses of MEL irradiation.

### 2.2. MEL Irradiation Using a Toxic Dose Induced Expressions of IL-1β, IL-6, and TNF-α in Cultured Human Skin Epidermal Keratinocytes

**Figure 2:**
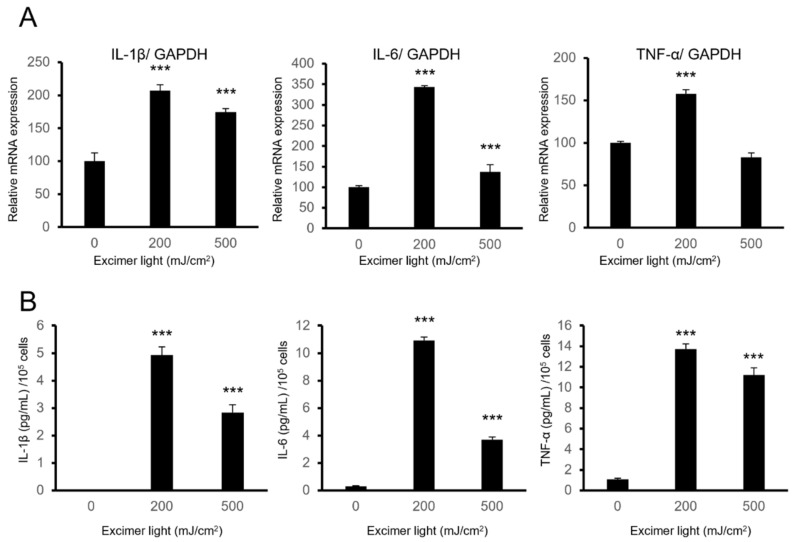
A toxic dose of monochromatic excimer light (MEL) irradiation induced IL-1β, IL-6, and TNF-α in PSVK1 keratinocytes. (**A**) Relative mRNA expression levels of IL-1β, IL-6, and TNF-α after 24 h irradiation with the indicated dose of MEL. (**B**) The protein expressions of IL-1β, IL-6, and TNF-α in cultured supernatants using an ELISA assay after 48 h of irradiation with the indicated dose of MEL. Data are shown as the means ± SD, *** *p* < 0.01, one-way analysis of variance followed by Dunnett’s test vs. 0 mJ/cm^2^.

Interleukin (IL)-1β, IL-6, and tumor necrosis factor alpha (TNF-α) are inflammatory cytokines, which are anti-melanogenic factors and are upregulated in the skin and sera in patients with vitiligo [28,29,30,31,32,33,34,35,36]. These cytokines may negatively affect therapeutic efficacies. To examine whether and how the high dose MEL irradiation affected the expression of these cytokines, mRNA expression and released protein amounts were evaluated after toxic doses of MEL irradiation in PSVK1 cells. Both mRNA (Figure 2A) and protein expression levels (Figure 2B) of IL-1β, IL-6, and TNF-α were significantly induced at toxic doses of 200 and 500 mJ/cm^2^ (Figure 2). These results suggested that expressions of IL-1β, IL-6, and TNF-α were upregulated when cells were exposed to toxic doses of MEL irradiation.

### 2.3. A Toxic Dose of MEL Irradiation Promoted ATP Release and Inflammasome Activation

**Figure 3:**
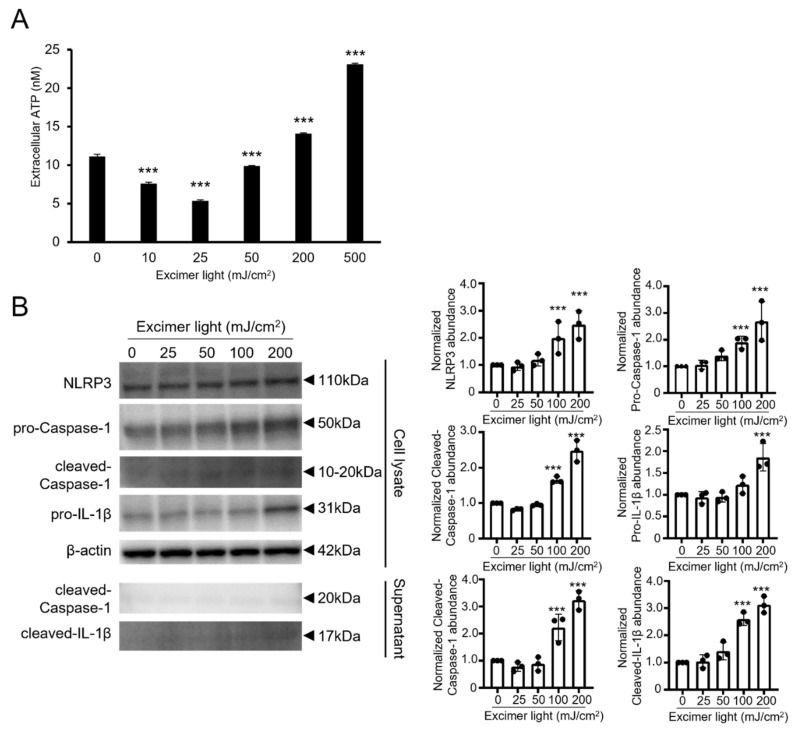
Monochromatic excimer light (MEL) irradiation altered extracellular ATP release and inflammasome activation in PSVK1 keratinocytes. (**A**) Extracellular ATP release was quantitated in the culture supernatant by an ATP quantitation assay after 2 h irradiation using the indicated dose of MEL. (**B**) The protein expressions of NLRP3, pro- and cleaved caspase-1, and pro-IL-1β in PSVK1 cell lysates and cleaved caspase-1 and IL-1β in the culture supernatant using Western blot analyses after 24 h irradiation with the indicated dose of MEL. The graph (right panel) shows averaged abundance normalized to β-actin by densitometry analysis of western blot bands. Data are shown as the means ± SD, *** *p* < 0.01 one-way analysis of variance followed by Dunnett’s test vs. 0 mJ/cm^2^.

In the skin during progressive vitiligo, IL-1β is increased with activation of NOD-like receptor family pyrin domain containing 3 (NLRP3) inflammasomes [36]. It was also reported that inflammasome activation led to the production of active IL-1β via the ATP/P2 × 7 axis in keratinocytes [37]. The high concentration of extracellular ATP induces both the production of reactive oxygen species and cell death in melanocytes. ATP release and inflammasome activation may, therefore, aggravate vitiligo and negatively affect the therapeutic efficacy. Hence, we next determined whether and how MEL irradiation affected the ATP release and inflammasome activation in PSVK1 cells. Extracellular ATP release was significantly increased using toxic doses of 200 and 500 mJ/cm^2^, while it was decreased using nontoxic doses of 10, 25, and 50 mJ/cm^2^ (Figure 3A). In addition, MEL irradiation increased the expression of the inflammasome components, NLRP3, and pro- and activated caspase-1 in a dose-dependent manner (Figure 3B). Consistent with results obtained from real-time PCR and enzyme-linked immunosorbent assays (ELISAs) (Figure 2), pro- and cleaved-IL-1β were detected using a toxic dose of 200 mJ/cm^2^.

### 2.4. A Lower Than Minimal Erythemal Dose Was Sufficient for Pigmentation in Guinea Pig Skin

**Figure 4:**
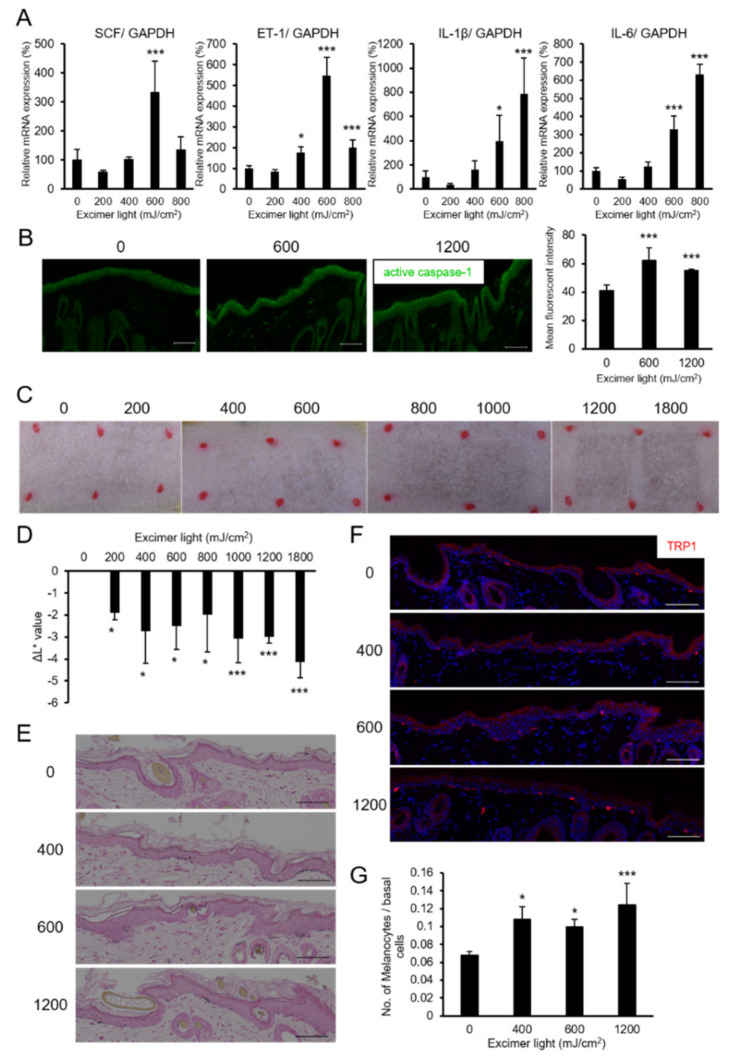
Monochromatic excimer light (MEL) irradiation induced skin pigmentation and melanocyte expansion in guinea pigs. (**A**) The mRNA levels of SCF, ET-1, IL-1β, and IL-6 were quantitated in the skin after 24 h irradiation with the indicated dose of MEL. (**B**) Immunofluorescence staining of active caspase-1 in the MEL-irradiated skin. Scale bars: 100 μm. The graph (**B**, right) shows the relative quantitation of active caspase-1 levels. (**C**) Representative examples of the skin after 14 days irradiation with the indicated dose (mJ/cm^2^) of MEL. (**D**) L* values of the irradiated skin on day 14. (**E**) Fontana–Masson melanin staining and (**F**) immunofluorescent staining for the melanocyte marker TRP1 at 14 days after irradiation using MEL with the indicated dose (mJ/cm^2^). Scale bars: 100 μm. (**G**) Quantitation of epidermal melanocytes from the skin of three guinea pigs for each condition. Data are shown as the means ± SD, * *p* < 0.05; *** *p* < 0.01; one-way analysis of variance followed by Dunnett’s test vs. 0 mJ/cm^2^.

The effect of MEL irradiation on melanogenesis was next verified in A-1 brownish guinea pigs in vivo. Three guinea pigs were treated with MEL at various doses in the corresponding areas, as shown in Figure 4C. The one minimal erythemal dose (1MED) of MEL in the A-1 guinea pigs was observed at 600 mJ/cm^2^ (Figure 4C). When MEL with approximately 1MED was singly irradiated, gene expressions of SCF and ET-1 increased with a peak at 600 mJ/cm^2^, whereas mRNA levels of IL-1β and IL-6 significantly increased with doses above 1MED (Figure 4A). MEL-irradiated skin at 1MED (600 mJ/cm^2^) or 2MED (1200 mJ/cm^2^) showed increased levels of active caspase-1 (Figure 4B). At 14 days after MEL irradiation, pigmentation was confirmed after MEL irradiation, even with 400 mJ/cm^2^ equivalent to 0.5MED, and the L values were significantly reduced (Figure 4C,D). In addition, it was confirmed that the amounts of melanin and numbers of melanocytes in the epidermal basal layer also increased (Figure 4E–G). Together, these results showed that pigmentation was sufficiently induced even at doses below 1MED.

### 2.5. A Lower Dose Was Effective for Pigmentation in the Rhododendrol-Treated Guinea Pig Skin

**Figure 5:**
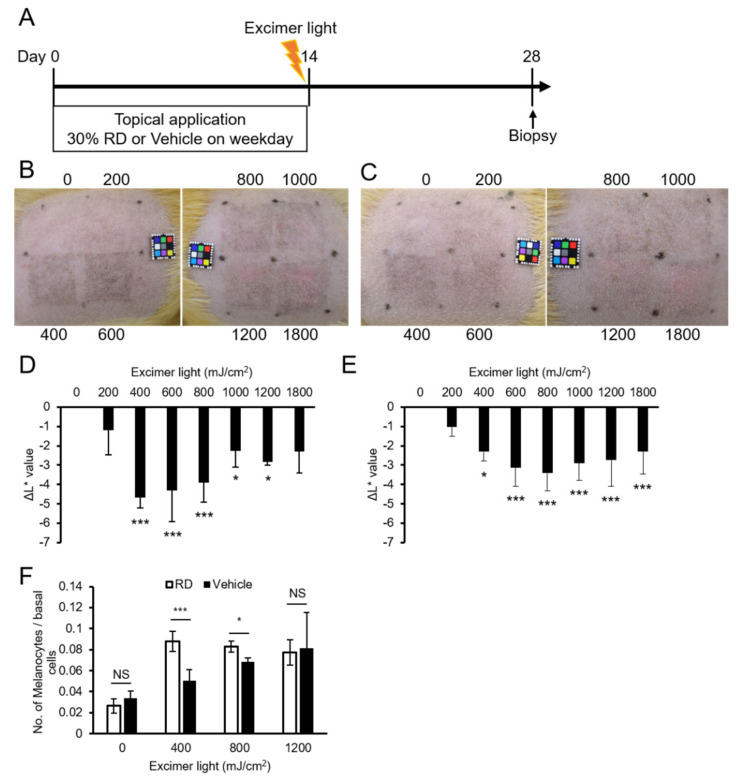
Monochromatic excimer light irradiation with a lower dose effectively induced skin pigmentation and melanocyte expansion in rhododendrol (RD)-treated guinea pigs. (**A**) Experimental design. (**B**) Representative examples of the skin of RD-treated guinea pigs on day 28. (**C**) Representative examples of the skin of the vehicle-applied guinea pigs on day 28. (**D**) ΔL* values of the skin of the RD-treated guinea pigs on day 28. (**E**) ΔL* values of the skin of the vehicle-treated guinea pigs on day 28. (**F**) Quantitation of epidermal melanocytes from the skin of three guinea pigs for each condition. Data are shown as the means ± SD, * *p* < 0.05; *** *p* < 0.01; Student’s *t*-test.

Rhododendrol (RD) induces chemical leukoderma in guinea pigs [38]. To understand the responsiveness of vitiligo-like skin to MEL, after continuous topical application with RD for 2 weeks, the back skin was irradiated with MEL, and the response to MEL irradiation was examined (Figure 5A). In control animals treated with solvent alone, pigmentation peaked at 800 mJ/cm^2^ (Figure 5B,D), whereas pigmentation was most prominent at a lower dose of 400 mJ/cm^2^ in skin treated with RD (Figure 5C,E). Furthermore, after skin sections were stained by anti-TRP1 antibody and the number of epidermal melanocytes was counted, the number of melanocytes increased significantly at 400 mJ/cm^2^ in skin treated with RD, when compared with the control. This change was not observed at high doses of 1200 mJ/cm^2^ (Figure 5F).

### 2.6. MEL Irradiation with a Suberythemal Dose Induced Skin Pigmentation and Melanocyte Recruitment in Mice

**Figure 6:**
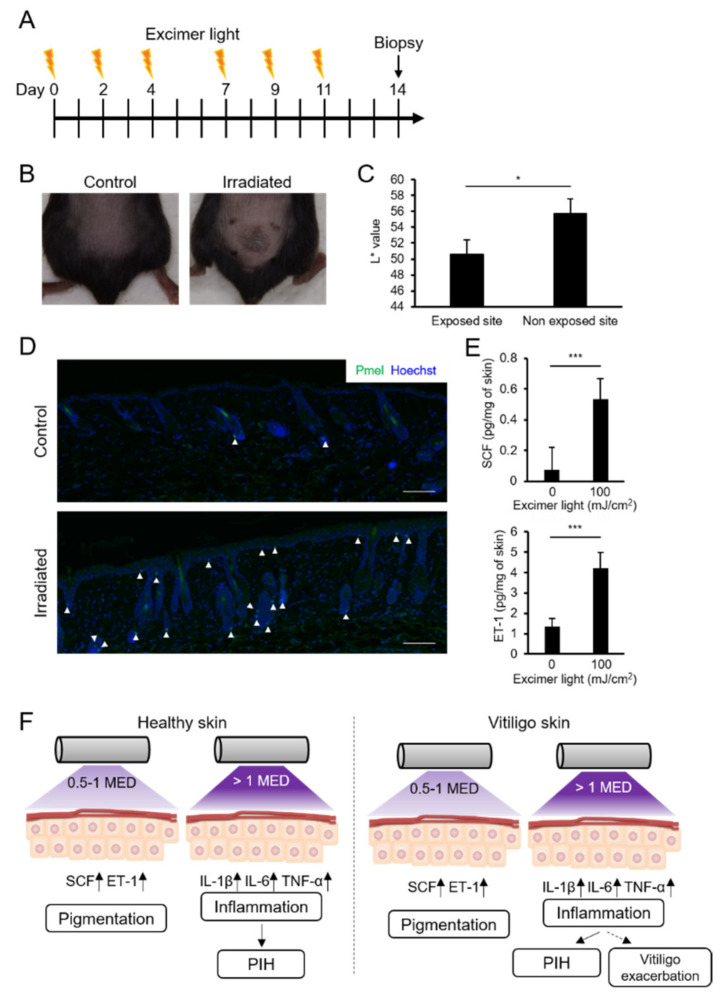
Monochromatic excimer light (MEL) irradiation with a suberythemal dose induced skin pigmentation and melanocyte expansion in mice. (**A**) Experimental design. (**B**) Representative examples of the skin of the irradiated and control mice on day 14. (**C**) L* values of the irradiated and non-irradiated skin on day 14. (**D**) Immunofluorescent staining for Pmel of the skin from the irradiated and control mice on day 14. Pmel-positive melanocytes (arrowhead) were induced in the skin irradiated by MEL. Scale bars: 100 μm. (**E**) The stem cell factor and endothelin-1 in irradiated and control skins were quantified using an ELISA assay. Data are shown as the means ± SD, * *p* < 0.05; *** *p* < 0.01; Student’s *t*-test. (**F**) Summary illustration of the possible response of MEL irradiation with suberythemal and the above erythemal doses involving pigmentation in healthy and vitiligo skin. MED, minimum erythema dose. PIH, post-inflammatory hyperpigmentation.

There are no melanocytes in the epidermis of mice, but it was reported that UVB-induced skin pigmentation and melanocyte migration [39,40]. The effect of MEL irradiation on melanogenesis was, therefore, investigated in C57BL/6 mice. The back skin was singly irradiated using MEL with a dose of 100, 300, or 600 mJ/cm^2^. There was no change in the appearance at 100 mJ/cm^2^, whereas the erythema reaction was observed at 300 and 600 mJ/cm^2^, and the epidermis was subsequently peeled off at 300 and 600 mJ/cm^2^. In this condition, MED should be >100 mJ/cm^2^. The back skin was, therefore, irradiated at 100 mJ/cm^2^, three times weekly for 2 weeks (Figure 6A). On day 14, the irradiated skin was pigmented (Figure 6B), and the L* value of the irradiated skin was significantly decreased (Figure 6C). Immunohistochemical staining of the epidermis revealed that Pmel-positive epidermal melanocytes were only observed in MEL-irradiated skin (Figure 6D). In addition, the expressions of mouse SCF and ET-1 were significantly increased in the skin on day 14 (Figure 6E). Together, these results showed that MEL irradiation with suberythemal doses induced melanocyte migration to the epidermis, which was sufficient for pigmentation (Figure 6F).

## 3. Discussion

There were three major findings from this study. First, the expressions of SCF, ET-1, and GPNMB in human keratinocytes were elevated by irradiation of MEL with a nontoxic dose; however, the expressions of IL-1β, IL-6, and TNF-α were elevated by irradiation of MEL with subtoxic and toxic doses. Second, in vivo skin pigmentation was induced even by suberythemal (0.5–1MED) irradiation of MEL in guinea pigs and mice. Third, irradiation with lower doses resulted in efficient pigmentation in a vitiligo-like model of guinea pigs.

In this study, the expressions of SCF, ET-1, and GPNMB were upregulated by MEL irradiation using nontoxic and suberythemal doses. In several reports, skin pigmentation was induced by UVB with 2MED [10,41]. Under these conditions, it was confirmed that the expressions of SCF and ET-1 were increased in humans. In the present study, we showed for the first time that SCF and ET-1 were induced even if the skin was irradiated by MEL with less than 1MED, and that skin pigmentation was accompanied by increased numbers of the melanocytes. In addition, to the best of our knowledge, for the first time, GPNMB in a human keratinocyte cell line PSVK1 was upregulated with low dose (50 mJ/cm^2^) irradiation with MEL. Our previous report suggested that the expression of GPNMB was abrogated in the vitiligo epidermis, suggesting its pathogenic involvement [42]. Effective use of UVB may thus lead to new treatments. To confirm this hypothesis in future studies, the dose setting is critically important, because excess UVB irradiation reduced GPNMB expression.

In the present study, with doses greater than 1MED, MEL irradiation was found to induce inflammatory cytokines. In healthy guinea pig skin, skin pigmentation was observed after the erythema reaction by MEL irradiation with ≥2MED and was considered to be post-inflammatory hyperpigmentation (PIH) (Supplementary Figure S1). The cause of PIH is not well-described, but various inflammatory cytokines (IL-1, IL-6, and TNF-α) and lipid mediators are reported to be involved [43]. PIH involves strong pigmentation, and hence, in the treatment of vitiligo, this is often problematic because the skin tone of PIH in repigmented or surrounding healthy skin is conspicuously different from the vitiligo lesion or original skin tone.

In addition, inflammatory cytokines induced by the MEL irradiation with 1MED were reported as aggravating factors and severity makers of vitiligo [33,36]. In particular, upregulated IL-1β in the skin and sera were found in the active progression of vitiligo and could play a central role in the cutaneous T cell response in vitiligo [36]. IL-1β is produced from pro-IL-1β by activated caspase-1, which is processed by inflammasomes [44]. It is known that UV-induced damage-associated molecular patterns, which contain extracellular ATP, promote inflammasome activation leading to IL-1β release. Ahn et al. [37] recently demonstrated that ATP-P2X7-driven inflammasome activation contributed to melanocyte death and CD8^+^ T cell trafficking via elevated expression of CXCL9. In the present study, MEL irradiation with toxic doses increased extracellular ATP levels; however, irradiation with nontoxic doses significantly reduced these levels in PSVK1 cells. Taken together, this study suggested that excess UVB irradiation may also be a major factor of inflammasome activation and vitiligo exacerbation, while low dose irradiation may suppress these processes.

In the present report, skin pigmentation accompanied with an increase in the number of epidermal melanocytes surprisingly occurred in skin irradiated with a lower dose of MEL in RD-treated guinea pigs, when compared with control animals. It was reported that the application of RD-induced melanocyte cell death and temporarily reduced the expressions of E-cadherin and DDR1, inducing melanocyte floating in a tyrosinase-dependent manner in mice [45,46]. In our previous report, the number of DOPA-positive melanocytes was greatly reduced by applying RD to guinea pigs [38]. Therefore, the number of epidermal melanocytes should decrease due to pretreatment with RD, but more effective pigmentation in RD-treated skin occurred using MEL irradiation with a lower dose. One possible reason was that the complementary molecular machinery needed to repopulate melanocytes in the epidermis may have already been activated. Further investigation is needed to understand this process and to expand our understating of human vitiligo and RD-induced leukoderma.

In conclusion, skin pigmentation was induced in healthy skin even at 0.5–1MED irradiation with MEL, and an inflammatory reaction occurred at a dose above 1MED, leading to dark, post-inflammatory pigmentation (Figure 6F). We propose that 0.5–1MED is the best dose for efficiently treating vitiligo without causing side effects. Although it was a small pilot study, a recent study reported that it was possible to regenerate skin pigment without side effects using half of the erythemic dose [47]. These results suggest that low-dose irradiation may provide adequate bio-stimulatory effects on melanin production and melanocyte differentiation in vitiligo lesions, without causing erythema and accidental burning. The therapeutic response of UVB treatment with suberythemic doses may, therefore, depend on the pathophysiology of each vitiligo patient, but it can be expected that the combination of low dose UVB irradiation and other therapies will enable more efficient treatment with fewer side effects. Nowadays, phototherapy is found to be the most efficient therapy for regimentation in vitiligo, and home phototherapy devices are also gaining popularity, the optimization for dosage, frequency, and safety is an urgent issue for vitiligo treatment. As well as the findings from in vitro and in vivo studies, there is still a need to design large multicentral randomized controlled clinical trial to gather more information about low-dose irradiation.

## 4. Materials and Methods

### 4.1. Cell Lines and Culture

Human PSVK1 keratinocytes, which are normal human keratinocytes immortalized by the pSV40 gene, were purchased from the Japanese Collection of Research Bioresources (ICRB Cell Bank, Osaka, Japan) and cultured in KBM-Gold keratinocyte basal medium supplemented with a KGM-Gold bullet kit (Lonza, Rockland, ME, USA).

### 4.2. Cell Treatment

PSVK1 cells were seeded into 35 mm dishes at a density of 3 × 105 cells/well for 24 h before treatment. Lids were removed and culture medium was replaced with phosphate-buffered saline (PBS) before MEL (TheraBeam UV308 mini; Ushio; Tokyo, Japan) irradiation. The peak wavelength of MEL is 308 nm, and TheraBeam UV308 is equipped with the cut-off filter for unnecessary short wavelength light. The irradiation illuminance is 25 mW/cm^2^. After irradiation using MEL at the indicated doses, the medium was replaced by fresh medium and then cells were incubated for different durations (24 h or 48 h). For quantitation of extracellular ATP, the PBS was not discarded, and fresh media was directly added to each dish after irradiation. The supernatants were then collected at 3 h after irradiation.

### 4.3. Cytotoxicity Assay

Cytotoxicity was measured using a Cytotoxicity LDH Assay kit-WST (Dojindo, Kumamoto, Japan) according to the manufacturer’s instructions. Briefly, 100 μL of cultured medium was transferred to each well of a clear 96-well plate. After the addition of 100 μL of working solution, the plate was incubated at room temperature for 30 min. Cell viability was then determined colorimetrically by measuring OD490 with a microplate reader (Model 550; Bio-Rad, Hercules, CA, USA).

### 4.4. RNA Isolation and qRT-PCR Analysis

Total RNA was extracted from cultured cells using a Maxwell 16 LEV simplyRNA tissue kit (Promega, Madison, WI, USA) following the manufacturer’s instructions. Total RNA (100 ng) was reverse transcribed using a ReverTra Ace qPCR RT master mix (Toyobo, Osaka, Japan). For extraction of total RNA from guinea pig skin, the skin tissue was immediately frozen in liquid nitrogen and homogenized using a bead mill (Multi-Beads Shocker; Yasui Kikai, Osaka, Japan). Total RNA was purified using a Maxwell 16 LEV RNA Tissue Kit (Promega) combined with TRIzol reagent (Thermo Fisher Scientific, Waltham, MA, USA) following the manufacturers’ instructions. RT-PCR was conducted using a QuantStudio5 Real-time PCR System (Applied Biosystems, Foster City, CA, USA). Reactions were run in triplicate using three independent experiments. The expression of the housekeeping gene, GAPDH, was used as an internal control to normalize variabilities in expression levels. The primers used for real-time PCR were as follows: human KITLG sense, 5’-TCAAGGACTTTGTAGTGGCATCTG-3’ and antisense, 5’-CTGCTACTGCTGTCATTCCTAAGG-3’; human EDN1 sense, 5’-GCAGAGAGCTGTCCAAGTCA-3’ and antisense, 5’- GTGCCCTTTTAACGGGGAGA-3’; human GPNMB sense, 5’-GCGAGATCACCCAGAACACA-3’ and antisense, 5’-AGAGCCAGGCTTGTGTCATC-3’; human GAPDH sense, 5’-GACAGTCAGCCGCATCTTCT-3’ and antisense, 5’-GCGCCCAATACGACCAAATC-3’; human IL1B sense, 5’- CAGAAGTACCTGAGCTCGCC-3’ and antisense, 5’-AGATTCGTAGCTGGATGCCG-3’; human IL6 sense, 5’-TGCAATAACCACCCCTGACC-3’ and antisense, 5’-ATTTGCCGAAGAGCCCTCAG-3’; human TNF sense, 5’-GTGACAAGCCTGTAGCCCAT-3’ and antisense, 5’-TCTGATGGCACCACCAACTG-3’; guinea pig Kitlg sense, 5’-AGAAAGGCCGAGCAGCAGAAT-3’ and antisense, 5’-TATAAGGCTCCAAAAGCAAAGCC-3’; guinea pig Edn1 sense, 5’-GGAAAGTGACCCACAACCGA-3’ and antisense, 5’-AGGGAGGGTTCTCTTGCTCT-3’; guinea pig Il1b sense, 5’-CCAGCGGATCTTCATTGCTC-3’ and antisense, 5’-TTTGCAGCTTGATCCCCTCA-3’; guinea pig Il6 sense, 5’-CTGCACAAGTTCAGCACGAC-3’ and antisense, 5’-GACAGCCTGGTAAACTCGCA-3’; and guinea pig GAPDH, sense, 5’-TTCTACCCACGGCAAGTTCC-3’ and antisense, 5’-CCAGCATCACCCCACTTGAT-3’.

### 4.5. ELISA Assay

The culture supernatants were collected, and the concentrations of SCF, ET-1, IL-1β, IL-6, and TNF-α were measured using specific ELISA kits (R&D systems, Minneapolis, MN, USA), according to the manufacturer’s instructions. The concentration of GPNMB in culture supernatants was measured using a GPNMB-specific ELISA kit (Abcam, Cambridge, MA, USA), according to the manufacturer’s instructions. Murine skin tissue was immediately frozen in liquid nitrogen and homogenized using a bead mill (Multi-Beads Shocker; Yasui Kikai, Osaka, Japan). Homogenized tissue was lysed by Lysis buffer 6 (R&D Systems), and the concentrations of SCF and ET-1 were measured using specific ELISA kits (R&D systems), according to the manufacturer’s instructions. The absorbance values at 450 nm were measured using a Model 680 Microplate Reader (Bio-Rad).

### 4.6. Western Blotting Analysis

For protein sample preparation, cell pellets, and supernatants were extracted as described previously [48], and 10 µg of extracted protein was used for Western blotting. The primary antibodies used at the following dilutions were as follows: anti-NLRP3 (Abcam) at 1:200; anti-cleaved caspase-1, anti-cleaved-IL-1β, and anti-β-actin (Cell Signaling Technology, Danvers, MA, USA) at 1:1000; anti-caspase-1 (Thermo Fisher Scientific) at 1:500; and anti-IL-1β (Abcam) at 0.2 μg/mL. We used anti-β-actin antibody as a loading control.

### 4.7. Quantification of Extracellular ATP

The culture supernatants were collected, and the concentration of extracellular ATP was measured using the ENLITEN ATP Assay System (Promega) according to the manufacturer’s protocol.

### 4.8. Animals and In Vivo Experimental Procedures

Twelve female 6-week-old A-1 brownish guinea pigs were purchased from Tokyo Laboratory Animals Science (Tokyo, Japan) and were maintained under specific pathogen-free conditions. In each guinea pig, eight areas (2 cm × 2 cm/area) of the dorsal skin were used after hair clipping by an electric shaver, and every experimental group contained three guinea pigs. The skin was irradiated with the indicated doses of MEL. A spectrophotometer (CM-26d; Konica Minolta; Tokyo, Japan) was used to evaluate brightness changes of the skin. In this study, the L* values (lightness) were used. Samples of treated skin were collected 1 day or 14 days after irradiation. In Figure 5, the animals were pretreated topically with 30% (*w/v*) rhododendrol (Kanebo Cosmetics, Tokyo, Japan) every weekday for 2 weeks to induce skin depigmentation. The control animals were treated topically with vehicle (50% (*v/v*)] ethanol/water) every weekday for 2 weeks. Twelve female C57BL/6 were purchased from CLEA Japan (Tokyo, Japan) and were maintained under specific pathogen-free conditions. The dorsal skin (1 cm × 1 cm/area) was used after hair clipping by an electric shaver. The dorsal skin was irradiated with the indicated dose of MEL three times weekly (on days 0, 2, and 4), and the skin was obtained on day 14. All animals were housed with free access to standard pellets and water. Every effort was made to minimize animal suffering. This study was conducted according to the Guiding Principles for the Care and Use of Laboratory Animals and approved by the Committee for Animal Experiments at Osaka City University (Permit No. 18042, Osaka, Japan).

### 4.9. Histology

Dorsal skin tissues from guinea pigs and mice were fixed in a 10% formalin neutral buffer solution (Wako Pure Chemicals, Osaka, Japan), embedded in paraffin, and sectioned on a microtome at a thickness of 5 μm. For Fontana–Masson staining, the sections were deparaffinized with xylene, rehydrated with a graded series of ethanol, and then subjected to Fontana-Masson staining using a Fontana–Masson Stain Kit (ab150669; Abcam) according to the manufacturer’s instructions. For TRP1 and Pmel immunohistochemical staining, 5 μm sections were incubated overnight at 4 °C with primary antibodies specific for TRP1 (HPA000937; 1:200 dilution; Sigma-Aldrich) or Pmel (ab137078; 1:500 dilution; Abcam), and then incubated with the secondary antibody (anti-rabbit IgG Alex Fluor 488 or 555; Thermo Fisher Scientific). Sections were counterstained with Hoechst 33,342 at a ratio of 1:500 (Thermo Fisher Scientific). For active caspase-1 staining, dorsal skin tissues from guinea pigs were fixed in a 4% paraformaldehyde phosphate buffer solution (Wako Pure Chemicals), embedded in OCT compound (Sakura Finetek, Tokyo, Japan), and sectioned by a microtome to a thickness of 10 μm. The frozen sections were stained using the FAM FLICA caspase-1 probe (ImmunoChemistry Technologies, Bloomington, MN, USA). The stained sections were visualized using a Biozero 8100 confocal microscope (Keyence, Osaka, Japan).

### 4.10. Statistical Analysis

One-way analysis of variance with a Dunnet’s test or an unpaired Student’s t-test (two-tailed) was used for statistical analyses. A value of *p* < 0.05 indicated statistical significance. All analyses were performed using EZR version 1.40 (Saitama Medical Center, Jichi Medical University, Saitama, Japan) and a graphical user interface for R version 3.5.2 (The R Foundation for Statistical Computing, Vienna, Austria).

## Data Availability

The data presented in this study are available on request from the corresponding author.

## Supplementary Materials

The following are available online at https://www.mdpi.com/article/10.3390/ijms221910409/s1.
