# Supplementary figures and images for: A Lower Irradiation Dose of 308 nm Monochromatic Excimer Light Might Be Sufficient for Vitiligo Treatment: A Novel Insight Gained from In Vitro and In Vivo Analyses
Source: Int J Mol Sci. 2021 Sep 27;22(19):10409. doi: 10.3390/ijms221910409 (PMC8508796; doi:10.3390/ijms221910409)

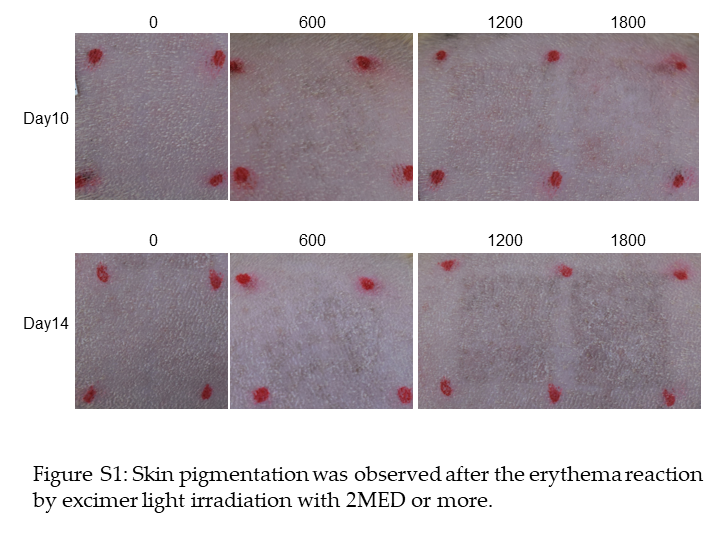

Supplement: Supplementary file 1 [file ijms-22-10409-s001.zip › ijms-1382082-supplementary.tif]
